# Fibrolamellar Hepatocellular Carcinoma: A Case Report of 25-Year Postoperative Survival

**DOI:** 10.7759/cureus.97131

**Published:** 2025-11-18

**Authors:** Kaito Fukuda, Moriyuki Kiyoshima, Iijima Tatsuo, Fuyo Yoshimi, Yusuke Kyoden

**Affiliations:** 1 Hepatobiliary and Pancreatic Surgery, The University of Tokyo, Bunkyo, JPN; 2 Surgery, Ibaraki Prefectural Central Hospital and Cancer Center, Kasama, JPN; 3 Thoracic Surgery, Ibaraki Prefectural Central Hospital and Cancer Center, Kasama, JPN; 4 Pathology, Ibaraki Prefectural Central Hospital and Cancer Center, Kasama, JPN

**Keywords:** ca125, fibrolamellar hepatocellular carcinoma, hepatocellular carcinoma, lymph node metastasis, repeated resection

## Abstract

Fibrolamellar hepatocellular carcinoma (FLHCC) is a rare hepatocellular carcinoma (HCC) variant. We report a 46-year-old patient with FLHCC who has achieved 25 years of postoperative survival, the longest reported to date. The patient underwent a right hepatic lobectomy, lymph node resection, and peritoneal dissemination removal. Over the years, he underwent seven additional surgeries for recurrences in various sites, including the intraperitoneal area, left lung, mediastinal lymph nodes, spleen, and transverse colon. To date, he has undergone a total of 14 surgeries, including eight for recurrences. FLHCC frequently recurs postoperatively despite its indolent nature. The patient’s cancer antigen 125 (CA125) levels correlated well with recurrence and treatment efficacy, confirming it to be a reliable tumor marker. Currently, the patient is undergoing radiation therapy for recurrence in the mediastinal lymph nodes. This case highlights the potential for long-term survival with aggressive surgical intervention and vigilant follow-up, emphasizing the importance of surgical resection and monitoring in FLHCC management.

## Introduction

Hepatocellular carcinoma (HCC) is the sixth most common cancer worldwide and the third leading cause of cancer-related death [1]. Its major risk factors include chronic hepatitis B and C infection, alcohol-related liver disease, and nonalcoholic fatty liver disease [2]. Prognosis depends on tumor stage and underlying liver function, and despite advances in systemic therapy and surgical techniques, long-term survival remains limited for most patients [3].

Fibrolamellar hepatocellular carcinoma (FLHCC) is a rare and unique histological variant of HCC that accounts for approximately 0.9-1% of all primary liver cancers [4]. Unlike conventional HCC, which typically arises in cirrhotic livers associated with viral hepatitis or chronic liver disease, FLHCC generally occurs in non-cirrhotic livers and affects adolescents and young adults without underlying hepatic dysfunction [4-6]. The disease is histologically characterized by large polygonal tumor cells with abundant eosinophilic cytoplasm arranged in lamellar fibrous bands [7,8]. Molecularly, FLHCC is distinguished by a recurrent chromosomal deletion on chromosome 19 resulting in a chimeric DNAJB1-PRKACA fusion gene, which drives aberrant activation of protein kinase A signaling and contributes to tumorigenesis [7,9,10].

Clinically, FLHCC often presents with vague symptoms such as abdominal pain, weight loss, or a palpable mass, leading to delayed diagnosis and large tumor burden at presentation [11,12]. Serum α-fetoprotein (AFP) levels are typically normal, complicating early detection and differentiation from other hepatic lesions [6]. While the tumor is considered biologically indolent compared to conventional HCC, it frequently recurs after curative resection, sometimes many years later, and can metastasize to unusual sites including lymph nodes, peritoneum, lungs, and mediastinum [11,13,14]. Despite the high recurrence rate, aggressive surgical resection remains the cornerstone of treatment and can result in prolonged survival, particularly when repeated resections are performed for recurrent disease [11,13,15].

Recent studies have also explored systemic therapies, including platinum-based agents, fluoropyrimidines, interferon-α, and more recently, molecular-targeted and immune checkpoint inhibitors, but their efficacy remains uncertain due to limited data [16-19]. Consequently, long-term survivors of FLHCC are exceedingly rare, and reports describing extended postoperative survival beyond two decades are exceptional.

Herein, we present a case of a patient diagnosed with FLHCC at 46 years of age who has survived for 25 years following the initial resection. To the best of our knowledge, the longest previously reported postoperative survival for FLHCC is 19 years; therefore, this case represents the longest postoperative survival to date [20]. Through repeated surgeries and vigilant tumor marker monitoring, the patient achieved sustained disease control despite multiple recurrences. This report highlights the importance of persistent surgical management, individualized treatment strategies, and continuous follow-up in improving long-term outcomes for patients with FLHCC.

## Case presentation

A 46-year-old man was diagnosed with FLHCC when he underwent right hepatic lobectomy, lymph node resection in the para-aorta, and peritoneal dissemination in the pouch of Douglas. As we previously described, he had survived several surgeries, including sigmoid colectomy, gynecomastectomy, and lymph node resection at that time [21].

He then underwent eight surgeries for recurrence in multiple sites, such as the intraperitoneal, left lung, lymph nodes in the mediastinum, spleen, and transverse colon. Table 1 summarizes his surgical treatments.

**Table 1:** List of operations conducted on the patient Surgeries for recurrence are indicated with a circle (○).

Operation number	Date	Type of operation	Operation for recurrence
1	1996/11	Exploratory laparotomy	
2	1997/11	Sigmoid colectomy	
3	1999/1	Right hepatic lobectomy, resection of the lymph node, and peritoneal dissemination in the pouch of Douglas	
4	1999/9	Gynecomastectomy	
5	1999/9	Resection of the lymph node	〇
6	2006/11	Mass resection	〇
7	2018/10	Mass resection and partial resection of the small intestine	〇
8	2018/10	Re-anastomosis for small intestinal anastomotic leak	
9	2019/5	Small intestinal fistula closure and bilobar valvuloplasty for small intestinal skin fistula	
10	2020/8	Robotic-assisted thoracic surgery left lower lobe lung resection	〇
11	2021/8	Splenectomy	〇
12	2022/8	Thoracoscopic mediastinal lymphadenectomy	〇
13	2023/1	Right hemicolectomy	〇
14	2023/7	Thoracoscopic resection of mediastinal lymph nodes	〇

**Figure 1:**
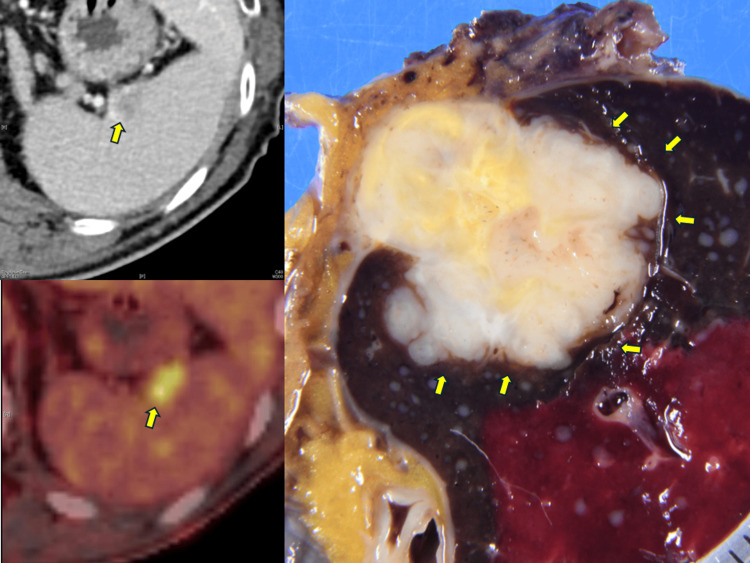
Imaging findings of recurrence in the spleen Contrast-enhanced computed tomography (CT) shows a hypodense lesion in the spleen (yellow arrow). Positron Emission Tomography (PET)-CT demonstrates fluorodeoxyglucose (FDG) uptake at the same site (yellow arrow). The cut surface of the resected specimen shows a white tumor nodule corresponding to the radiologic findings (yellow arrows).

The contrast-enhanced computed tomography (CT) and positron emission tomography (PET) imaging findings before the splenectomy and the resected specimen are shown in Figure 1.

**Figure 2:**
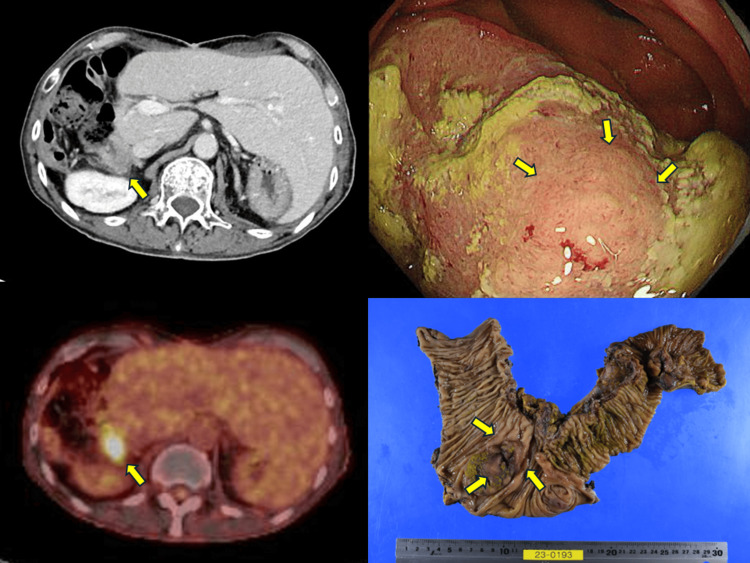
Imaging findings of recurrence in the colon Contrast-enhanced computed tomography (CT) shows wall thickening of the ascending colon (yellow arrow), and positron emission tomography (PET)-CT demonstrates fluorodeoxyglucose (FDG) uptake (yellow arrow). Endoscopy reveals a mucosal mass (yellow arrows). The resected specimen confirmed that the tumor was located primarily within the mucosal layer (yellow arrows).

The contrast-enhanced CT, PET, and endoscopic imaging findings before the colectomy and the resected specimen are shown in Figure 2.

**Figure 3:**
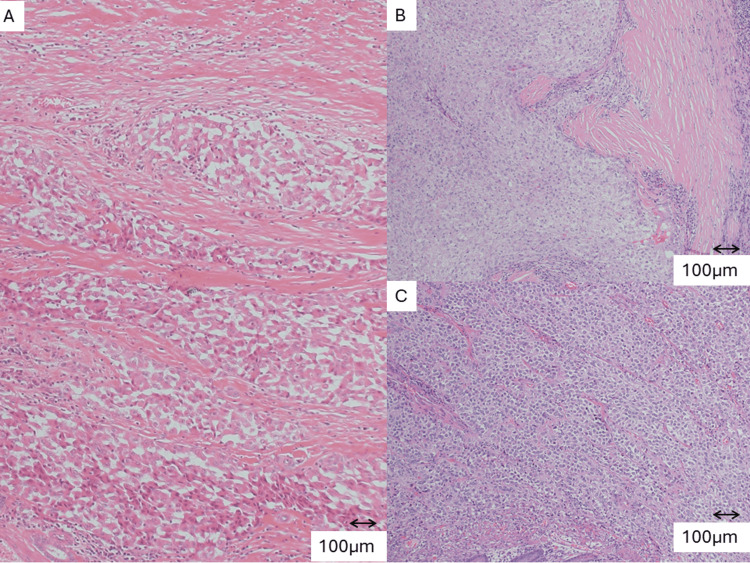
Pathological findings (H&E, ×100) Hematoxylin and eosin (H&E) staining demonstrates typical fibrolamellar carcinoma with lamellar fibrosis. (A) Liver (initial hepatectomy specimen); (B) Colon recurrence; (C) Splenic recurrence. Lamellar fibrosis and eosinophilic tumor cells are observed in all specimens. Scale bar = 100 µm.

The pathological images are shown in Figure 3.

Eosinophilic tumor cells in the lamellar fibrosis were observed in the liver specimen from the initial surgery, as well as in the spleen and colon specimens.

**Figure 4:**
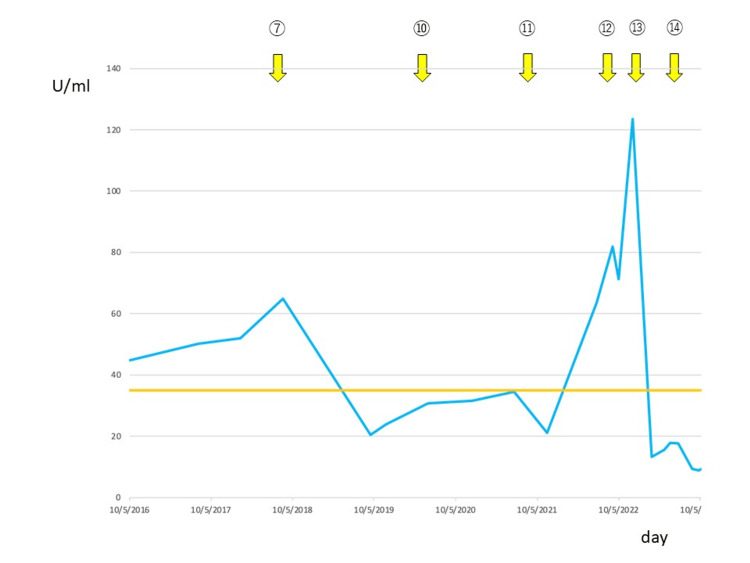
The trend of CA125 levels Changes in cancer antigen 125 (CA125) levels over time from the 7th to the 14th surgery. Yellow arrows indicate the timing of each surgery. CA125 elevation correlated with recurrence and decreased after tumor resection.

Figure 4 demonstrates the trends of cancer antigen 125 (CA125) and treatments.

The value of CA125 correlated well with recurrence and treatment efficacy. A detailed analysis of his surgeries is presented in Table 2.

**Table 2:** Analysis of surgical interventions in the patient and corresponding trends in CA125 levels CA125, cancer antigen 125; PALN, para-aortic lymph node; LN, lymph node

Operation number	Date	Interval until reoperation for recurrence (months)	Organs operated upon	Value of CA125 before surgery (U/mL)	Value of CA125 after surgery (U/mL)
3	26-01-1999	-	Liver, PALN, Dissemination	3780	92
5	27-09-1999	8	LN	126	15
6	10-11-2006	87	Dissemination (Abdominal wall)	30.4	10.6
7	02-10-2018	145	Dissemination with small intestine	64.8	20.5
10	06-08-2020	22	Lung	30.8	31.6
11	24-08-2021	13	Spleen	34.4	21.1
12	16-08-2022	12	LNs in mediastinal	63.6	81.8
13	16-01-2023	5	Colon	123.5	13.3
14	20-07-2023	6	LNs in mediastinal	17.7	9.3

The CA125 values before and after each surgery are documented, highlighting the correlation between the CA125 levels, recurrence, and treatment efficacy. CA125 values were generally elevated before recurrence and tended to decrease post-surgery. Notably, a significant decrease in CA125 values was observed in the initial surgery (operation number 3). For operation number 10, there was no significant change in CA125 values before and after surgery, with a slight increase observed post-surgery. Similarly, the operation number 12 showed an increase in CA125 values after the surgery, which might be indicative of disease progression or other contributing factors.

The patient is now under radiation therapy for recurrence in the mediastinal lymph nodes because of massive invasion into the esophagus, bronchus, and aorta. To date, the patient has undergone a total of 14 surgeries, including eight surgeries for recurrence. He remains alive and well for 25 years post the initial resection.

## Discussion

To the best of our knowledge, the longest previously reported postoperative survival is 19 years; thus, this case represents the longest documented survival to date [20].

We experienced a long-term survivor of FLHCC following aggressive and multiple surgical interventions. FLHCC, first described by Edmondson in 1956, accounts for approximately 0.9% of all HCC cases [4]. The average age of patients is 39 years [4], with an equal gender distribution. FLHCC is characterized as an indolent tumor with a favorable prognosis whose five-year survival rate ranges from 32% to 76% [4,11,20,22]. In particular, patients treated with hepatectomy have excellent long-term survival, with five-year overall survival reaching 70% but 50-80% of them relapse [6]. Undoubtedly, hepatic resection for liver mass is the foremost treatment for FLHCC; however, the treatment policy for concomitant extrahepatic lesions is unclear. Several factors, such as tumor, node, and metastasis staging components, multiple tumors, vascular invasion, lymph node status, and distant metastasis during the initial surgery, have been demonstrated to be negative prognostic factors in previous surgical series [6,13]. In our experience, extrahepatic lesions, such as distant lymph node metastasis or disseminated nodules, have not been contraindications for surgery, but the location and timing of the metastasis may influence the treatment policy.

FLHCC frequently recurs even after complete surgical resection despite its indolent tumor biology. Various recurrence sites have been reported, including the chest wall, peritoneum, stomach, diaphragm, adrenal glands, and pancreas [13,14,23]. In our case, we experienced uncommon metastatic sites, including the spleen and ascending colon. To the best of our knowledge, no previous reports have described resectable metastases occurring at such unusual sites, including the spleen and ascending colon. FLHCC can recur at any site unexpectedly.

Recent reports have discussed systemic therapy [16] and immune checkpoint inhibitors [17]. However, as Glavas et al. mentions, surgery is the first-choice treatment for FLHCC, and appropriate treatments for recurrence remain unclear due to the lack of information [18]. The ideal treatment for FLHCC recurrences seems to be surgical resection [5,12]. Several authors have reported aggressive surgical treatments even for recurrence with favorable outcomes [11,13,15]. Glavas et al. [18] noted that FLHCC has high recurrence rates and poor long-term outcomes, emphasizing the need for more specific treatments for advanced and recurrent disease. Our aggressive surgical approach is also acceptable for managing FLHCC recurrences.

FLHCC presents distinct oncological features compared to conventional HCC. In this case, resection was performed at intervals as short as five months (Table 2). No instances of local recurrence or unresectable peritoneal dissemination were observed up until the latest recurrence in the paraaortic lymph nodes despite undergoing multiple surgeries within a short period. This second recurrence in the paraaortic lesion finally hampered an attempt at resection, but active management of disease relapse requires early detection [11]. Vigilant and careful follow-up is warranted for early disease detection; however, the optimal timing of resection for recurrent FLHCC requires further investigation.

CA125 is a reliable tumor marker in this case and is frequently beneficial in malignant ovarian serous tumors [24], and has been reported to be helpful in the diagnosis of HCC [25]. As is often the case, typical tumor marker elevations, such as AFP and prothrombin induced by vitamin K absence-II, are not observed. In this patient, AFP remained consistently within the normal range throughout the clinical course and showed no correlation with recurrence. Therefore, serial AFP values were not included because they did not contribute to clinical decision-making. Several studies reported on the use of other tumor markers, but they remain inconclusive due to limited reported cases [8,26-28]. Further investigation is required as the molecular role of CA125 in FLHCC is not yet well understood. However, in HCC, CA125 has been shown to be involved in cell proliferation, adhesion, and migration, suggesting a potential role in tumor progression and metastasis. In clinical practice, CA125 levels can also be influenced by non-tumor conditions such as inflammation, infection, ascites, or postoperative changes [29]. In this patient, however, CA125 elevation consistently preceded radiologic recurrence and decreased after resection, suggesting that the fluctuations were more likely associated with tumor activity rather than nonspecific inflammatory responses.

Few effective chemotherapies for FLHCC exist beyond surgical resection, with some reports indicating platinum-based agents, fluoropyrimidines, and interferon-α (INF-α) [19]. Systemic therapies, although varied, have shown some promise, with factors like high baseline neutrophil-to-lymphocyte ratio (NLR) and radiomic features being associated with prognosis. In a study analyzing data from 23 patients, treatments included sorafenib, capecitabine, and interferon, with a median overall survival of 26.7 months [16]. Genomically, FLHCC is defined by a 400-kB deletion on chromosome 19, forming the DNAJB1-PRKACA fusion protein. This distinct molecular entity may result in novel therapeutics in the future [7]. Targeted therapies, specifically designed to inhibit the DNAJB1-PRKACA fusion protein, offer a promising avenue for treatment [9]. Additionally, combination therapies integrating kinase inhibitors with immune checkpoint inhibitors may enhance therapeutic efficacy and overcome resistance mechanisms [10,30]. Furthermore, ongoing research into the molecular pathways influenced by the DNAJB1-PRKACA fusion protein could reveal additional therapeutic targets, such as specific downstream signaling molecules and transcription factors. Personalized medicine approaches, leveraging genomic and proteomic profiling of individual tumors, might optimize treatment strategies and improve outcomes for FLHCC patients. This highlights the need for continued research and clinical trials to fully explore and validate these emerging therapeutic options.

We must mention the disadvantages of multiple surgeries. In our case, two surgeries were performed for postoperative complications. Especially, the enterocutaneous fistula was intractable and was treated with the assistance of a plastic surgeon. Aggressive surgical policy involves not only technical difficulties but also meticulous postoperative complication management. This included infection monitoring, nutritional support, and physical therapy. These complications and repeated surgeries significantly affected the patient's quality of life, leading to prolonged hospital stays and multiple recovery periods [31,32]. Despite these challenges, the patient maintained a good performance status throughout his clinical course. He continued his usual daily activities and remained socially and occupationally active. Importantly, each surgical intervention was performed at the patient’s strong request, reflecting his motivation to pursue active treatment rather than psychological distress or external pressure. Although he experienced chronic diarrhea requiring medical therapy, symptoms remained well controlled, allowing preservation of social functioning and overall quality of life.

This study has several limitations. Genetic analysis, including confirmation of the DNAJB1-PRKACA fusion gene, was not performed because of financial constraints. Future investigations incorporating comprehensive molecular profiling may help clarify the genetic background of long-term survivors and identify candidates for targeted therapies. Furthermore, multicenter collaborations will be essential to establish standardized management strategies for recurrent FLHCC.

Although FLHCC is generally considered an indolent tumor, it frequently recurs, and resectability varies among institutions and surgeons. In this case, repeated resections were feasible and effective even when metastases occurred at unusual sites. These findings suggest that repeated surgical resection combined with vigilant surveillance may contribute to prolonged survival in appropriately selected patients. While this report describes a single case and its generalizability is limited, further multi-center studies are required to determine whether this strategy can be incorporated into broader clinical practice.

## Conclusions

This case demonstrates that prolonged survival in FLHCC is achievable through repeated surgical resection of recurrent disease. Over 25 years, our patient underwent multiple metastasectomies at several uncommon sites, resulting in durable disease control and the longest postoperative survival reported to date. In addition, CA125 served as a useful adjunctive marker for early recurrence when conventional tumor markers remained within normal ranges. Further studies are needed to clarify the clinical utility of CA125 and to establish optimal surveillance and treatment strategies for patients with recurrent FLHCC.
